# Positive impacts of a covered veranda on broiler chicken welfare

**DOI:** 10.1016/j.psj.2025.105347

**Published:** 2025-05-26

**Authors:** F. Mocz, J-P. Moysan, L. Warin, A. Keita, V. Michel, M. Guinebretière

**Affiliations:** a Epidemiology, Health and Welfare Unit, Ploufragan-Plouzané-Niort Laboratory, French Agency for Food, Environmental and Occupational Health & Safety (ANSES), Anses, BP 53 Route de Beaucemaine, Ploufragan, 22440, France; b Technical Institute for Poultry (ITAVI), Nouzilly, 37380, France; c Avian Experimental Unit, Ploufragan-Plouzané-Niort Laboratory, French Agency for Food, Environmental and Occupational Health & Safety (ANSES), Ploufragan, France; d Direction of Strategy and Programmes, French Agency for Food, Environmental and Occupational Health & Safety (ANSES), Maisons-Alfort, France

**Keywords:** Welfare, Broiler, Health, Behavior, Covered veranda

## Abstract

In standard broiler production systems, birds do not have outdoor access and spend their entire lives in the same environment. A covered veranda can improve broilers’ living conditions by providing access to different environments and to areas with varying space availability (i.e. different stocking densities), while also allowing them to experience outdoor sensory stimulations and protecting them from predators and the risk of disease. This study aimed to evaluate the welfare and health of broilers with access to a covered veranda. Redbro broilers were randomly assigned to six identical enriched rooms (with elevated platform and alfalfa bales), each containing 2,478 birds (final stocking density of 26 kg/m²). Three rooms had access to a covered veranda from 22 days of age (D22), while the other three had no such access (control). Footpad dermatitis, hock burns and gait were assessed on D36. We also measured activity levels (D34 and D37), use of the covered veranda (from D22 to slaughter), litter quality (D34 and D37), as well as feed and water consumption throughout the rearing period. Mortality and body weight were also monitored. Access to a covered veranda did not affect mortality, body weight, litter quality, or feed and water consumption. None of the broilers from either treatment group exhibited gait issues or footpad dermatitis. However, the prevalence of hock burns was lower in the broilers having access to a covered veranda. Additionally, more active behaviors were observed among broilers from the covered veranda group than from the control group. The covered verandas were frequently used, with usage increasing as the broilers aged. Within a day, use was more frequent in the morning and decreased thereafter. Providing access to a covered veranda improved bird welfare without negatively impacting zootechnical indicators.

## Introduction

In general, broilers raised in conventional systems are kept indoors, with or without access to various enrichments (perches, pecking objects, elevated platforms, etc.) depending on national or private specifications. In the absence of enrichment, or when enrichment is provided in insufficient quantities, these systems may impair the expression of specific behaviours such as foraging, perching, dustbathing, exploring or sunbathing (EFSA, 2023). While access to an outdoor range can grant the birds the conditions needed to fulfil their behavioural needs and possibly improve their welfare (El-Deek and El-Sabrout, 2019), it may also introduce risks, like predator attacks or an increase in disease (e.g. avian influenza). A covered veranda can grant some of the advantages of an outdoor range for the birds while protecting them from predators and greater risks of disease. For instance, covered verandas are particularly valuable on free-range farms during periods when access to outdoor areas is restricted due to disease risk, as they help prevent stressful complete confinement of the birds. Although some poultry farms (those that raise laying hens, broilers, or turkeys) do not provide access to outdoor ranges, they do offer access to a covered veranda, the exact number of such farms remains difficult to quantify in France.

A covered veranda is a non-heated area adjacent to the barn, accessible to the birds via popholes in the side of the barn and partially exposed to outdoor environmental conditions (EFSA, 2023). It has a roof, is separated from the outdoors by wire mesh (and possibly windbreakers), and the floor is covered with litter. The covered veranda gives birds extra space (although only if its surface is not included in the stocking density calculation) and the choice of accessing daylight and outdoor conditions (e.g. fresh air, different temperatures). The opportunity of choosing between an indoor and outdoor environment may in itself positively impact broiler welfare, as making choices enables them to exercise control, thereby increasing their motivation and wellbeing (Englund and Cronin, 2023).

Although some previous studies examining different rearing systems included outdoor access via a covered veranda (Bergmann et al., 2016, 2017; Dawkins et al., 2003; Gocsik et al., 2016; Goransson et al., 2021; Louton et al., 2019), no studies have investigated the impact of covered verandas per se on the welfare of broiler chickens. The goal of the present study was therefore to assess the use of the covered veranda (without outdoor access) by Redbro broilers, their locomotor activity in this area and the effects of the veranda on the birds’ health and welfare. For this evaluation, the focus was on animal-based measures (footpad dermatitis, hock burns, gait, behaviors), on zootechnical indicators (mortality, feed and water intake, body weight) and on litter quality.

## Material and methods

### Ethics statement

The housing, management and experimental procedures all complied with European legislation on the protection of animals used for scientific purposes (EU Directive 2010/63/EU), and were approved by ANSES’s ethical committee (D-22-745-1, Opinion no. 2023-02-14-09).

### Housing and experimental scheme

**Fig. 1:**
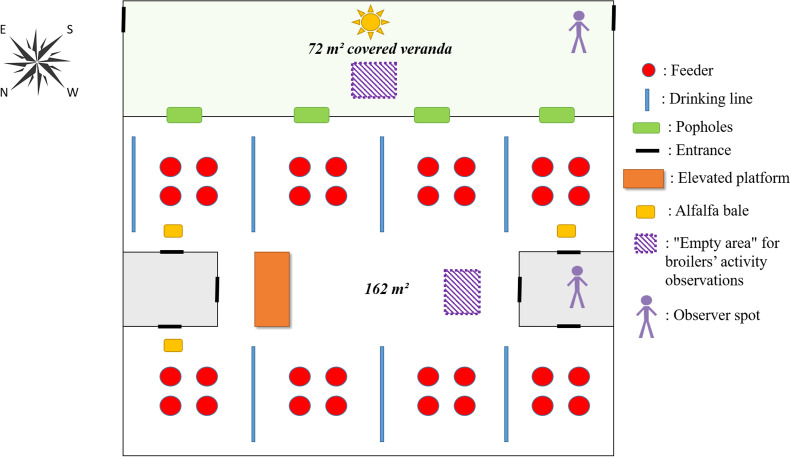
Drawing of one experimental room (popholes open 24/24 h from 22 days of age for CV treatment group; popholes permanently closed for C treatment group).

**Fig. A1:**
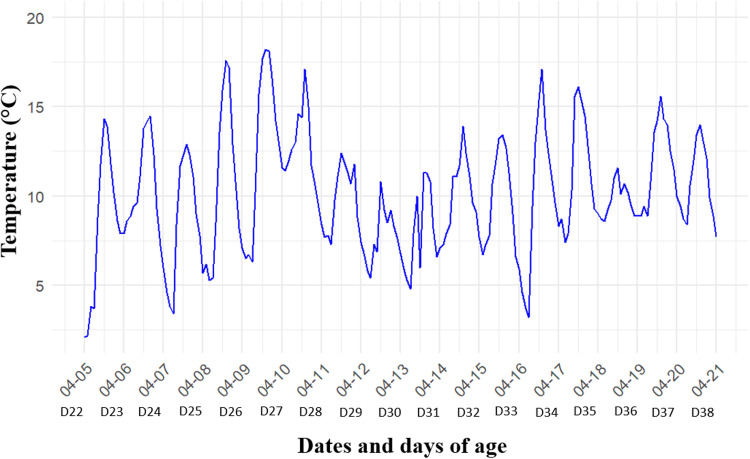
Temperature evolution every two hours during the experimental period (from 05-04-2023 to 21-04-2023) when broilers from the CV treatment group had access to the covered verandas (from 22 to 38 days of age). Data from local weather station (approximately 5 km from the experimental facilities).

A total of 14,872 one-day-old Redbro broilers (49 g/d, from 31-week-old breeders) were delivered in March 2023 and divided randomly between six identical 162-m² rooms in the ANSES Avian experimental building (Ploufragan, France). A group of 2,478 chicks were placed in each room to reach a final density of 26 kg/m² when slaughtered at 1.9 kg and 38 days of age (the covered veranda surface was not included as an available area for stocking density calculation). All the rooms had natural light through eight windows (90 cm x 30 cm) opened from 08:00 to 19:00. The natural light was supplemented by artificial light guaranteeing 30 lux for 18 h per day from 6 days of age (from 06:00 to 23:59). In each room, all the birds had access to one elevated platform of 2 m² with access ramps of 1 m² from both sides (4 m² of platform in total) and three alfalfa bales (each 20 kg, 30 cm high, 50 cm long, 30 cm wide). Sawdust litter was scattered in each room following standard management practices (1.2 kg/m²). All the chickens had permanent ad libitum access to feed and water. Three rooms had four popholes (46 cm high, 100 cm long) open 24/24 h to allow broilers access to a covered veranda of 72 m² (CV rooms) (Fig. 1) from 22 days of age, whereas the other three rooms had closed popholes (C rooms). The floor of the covered verandas was concrete, covered with straw pellets (2 kg/m²), and the side facing the outdoor area was made of wire mesh. The roof was equipped with an anti-drip film to prevent condensation and water dripping onto the broilers. Windbreaker netting was attached to the wire mesh to protect the broilers from wind and rain. The local temperatures, according to weather records, ranged from 1.8°C to 18.6°C (details in Appendix Fig. A1) during the period when the birds had access to the covered verandas.

### Data collection

***Body weight and mortality.*** A total of 150 randomly selected birds per treatment group (50 per room, sex balanced as soon as feasible: from D29) were manually and individually weighed on D8, D15, D22, D29 and D36. The number of dead broilers per room was recorded daily (found dead or culled if needed to avoid the birds’ suffering).

***Leg health and gait score.*** On D36, the broilers selected for weighing were also assessed for gait score, along with the presence and severity of footpad dermatitis and hock burns by two trained assessors. Scoring ranged from 0 to 2 points for each criterion (Table 1). After weighing, the broiler was carried by a person who presented both legs to the observer. The observer examined both legs and scored the condition of the worst one. The bird was then returned to the group, and the second observer recorded the gait score while observing the bird from behind, and gently encouraged it to walk with a stick if needed.

**Table 1:** Scoring of footpad dermatitis, hock burns and gait score (Guinebretière et al., 2024).

	Score 0	Score 1	Score 2
Footpad dermatitis	None or slight, very superficial lesions, slight discoloration over a limited area, slight hyperkeratosis or scarred skin	Moderate to severe discoloration, superficial lesion, darkened papillae	Severe dermatitis ulcers or scabs of significant size, signs of bleeding or severely swollen foot pads
Hock burns			
Gait score	Normal gait, agile, with or without imbalance	Broiler walks more than 1.50 m but has difficulty in walking; limps	Broiler walks less than 1.50 m, and/or is severely lame, preventing movement

***Litter quality.*** On D34 and D37, the litter quality of each room was assessed by a single assessor in three areas (in the middle of the room, under drinkers and between feeders) giving 3 scores per room, using the Welfare Quality protocol (Welfare Quality®, 2009): score 0= completely dry and flaky, score 1= dry, but not easy to move with foot, score 2= leaves imprint of foot and will form a ball if compacted, but ball does not stay together well, score 3= sticks to boots and sticks readily in ball if compacted, score 4= sticks to boots once the cap or compacted crust is broken.

***Activity level and resting behaviors.*** The behavior of the broilers was recorded at 34 and 37 days of age by a single observer. Following a 1 min period of acclimatisation to the observer's presence, the observations were recorded over 3 min. Every occurrence of birds walking and running (definitions in Table 2, adapted from Lourenco da Silva et al. (2021)) in a 2-m² “empty” observation area (far from feeders, drinkers and enrichments) inside the rooms and in the covered veranda of the CV treatment group (see Fig. 1) were noted, without individual identification. The observer also counted the total number of broilers — including birds lying down — in the 2-m² area under observation before and after each 3-min observation period using the scan sampling method.

**Table 2:** Experimental ethogram adapted from (Lourenco da Silva et al. 2021).

Behavior	Definition
*Walking*	Broiler moves at a slow pace
*Running*	Broiler moves at a fast pace and occasionally flaps its wings
*Lying/resting*	Broiler lies on the litter with its head resting on the ground or erect; eyes may be open or shut

Observation was repeated 2 to 4 times in each room and covered verandas on D34 and D37. Due to technical constraints, the number of repetitions per room and covered veranda varied between D34 and D37 (Table 3). The observations took place between 09:20 and 15:45 and were evenly distributed between the two treatment groups over both the morning and afternoon, ensuring that neither treatment was assessed only during a specific time period.

**Table 3:** Number of behavioral observation sessions in the rooms of both treatment groups and covered verandas on D34 and D37.

	D34	D37
Indoors	4 times in each of the CV and C rooms	3 times in each of the CV and C rooms
Covered verandas	2 times in each of the covered verandas	2 times in each of the covered verandas

***Use of the covered verandas.*** Video recordings were studied by a single observer to assess use of the covered verandas. The videorecorded surface area was half the size of the covered veranda (36 m² from the popholes to the middle of the covered veranda’s length). The number of chickens was counted seven times a day, using scan sampling, between 22 and 37 days of age at 08:00, 10:00, 12:00, 14:00, 16:00, 18:00 and 20:00.

### Statistical analysis

Data were pre-processed and statistical analyses performed using R software v. 4.0.3 and RStudio (R Core Team, 2023). The statistical unit was the room. Initially, several statistical models were tested for each data set, incorporating different variables as fixed or random effects. Final models were selected based on their stability (e.g., AIC, BIC), and are described as follows.

After visually validating the distributions of the model residuals for normality, a linear model was used to analyse the variance in body weight data. The final model for individual weight data included treatment and the interaction between treatment and bird age as fixed effects.

Generalised negative binomial regression models were used to analyse the daily number of dead chickens (found dead and culled) from D1 to D36 (“total mortality”) and occurring only after D22 (from the day of access to covered verandas in CV rooms). For mortality from D1 to D36, the final model included room and age as random effects, with treatment as a fixed effect. The final model analysing mortality after D22 incorporated treatment as a fixed effect and no random effects.

To analyse the variables impacting the number of broilers counted in the covered verandas, a generalised linear mixed model (GLMM) with a negative binomial distribution was used, with age and hour as fixed effects and the room as a random effect.

To quantify the walking and running behaviors, standardised occurrence rates were calculated by dividing the total occurrences of walking and running observed during the 3-min observation period by the average number of birds present in the 2 m² observation area during the same 3 min of observation (averaging the number of birds counted in the area before and after the 3 min of observation), and then multiplying by 100 to express the rate as occurrences per 100 broilers. In addition, the percentage of broilers lying down in the area was noted before and after the 3 min of observation. These standardised occurrence rates for walking and running, and the percentage of birds lying down, were then analysed using linear models. The models included location (inside CV rooms, inside C rooms, or covered verandas), age and the interaction between age and location as fixed effects. Running, walking and lying behaviors were analysed in three different ways:

- Comparison of behaviors observed inside the CV rooms with those noted inside the C rooms: “CV vs. C, Indoor activity”.
- Comparison of behaviors observed in the covered verandas with those observed inside the CV rooms: “Indoor vs. outdoor, CV treatment group”.
- Comparison of the two treatments, using the average activity level of the CV treatment group (mean of behaviors noted in the covered verandas and inside the CV rooms) versus the behaviors of the control group (behaviors noted inside the C rooms): “CV vs. C”.

In the absence of normality of the residuals, the water and feed consumption and the litter quality were analysed with Mann-Whitney non-parametric tests to compare treatments. Chi-square tests for each severity score were used to compare gait score, footpad dermatitis and hock burn data for each treatment group.

For all analyses, significant interactions were followed by post-hoc analyses of estimated marginal means with a Tukey adjustment. Differences were considered significant when *P* ≤ 0.05.

## Results

### Mortality and body weight

The total mortality was 3.90 % (standard deviation: 1.37 %) for the CV treatment group, including 3.10 % (1.43 %) in the first 8 days of age, and 7.12 % (5.05 %) for the C treatment group, including 6.07 % (4.80 %) in the first 8 days of age. However, no significant effect of treatment was found on the total mortality (p = 0.15) or specifically after D22 (0.29 % (0.08 %) for the CV group when covered verandas were accessible vs. 0.40 % (0.14 %) for the C group (p = 0.58).

There was no significant difference between treatment groups in terms of body weight, regardless of age (p = 0.88) (Table 4).

**Table 4:** Mean weights (g) (±standard deviations) of broilers from both treatment groups, individually weighed on D8, D15, D22, D29 and D36. Group C=Control group; Group CV=Group with covered veranda access.

	D8	D15	D22	D29	D36
Group C	166.7 (18.7)	414.4 (42.4)	816.3 (79.9)	1270.0 (143.1)	1815.9 (237.4)
Group CV	164.4 (17.0)	408.9 (41.9)	805.1 (73.7)	1269.6 (153.8)	1825.0 (225.1)

### Leg health and gait score

No gait issues or signs of footpad dermatitis (score 0) were observed in any of the birds assessed on D36, in either group, making statistical analysis unnecessary. However, the prevalence of moderate hock lesions (score 1) was higher in the C treatment group than in the CV treatment group (15.3 % vs. 1.3 %, respectively; p < 0.001). No broilers were observed with the worst score (score 2) for hock burns.

### Litter quality, water and feed consumption

There was no effect of treatment on the litter quality on D34 (average CV score: 0.67; average C score: 0.33; p=0.53) or on D37 (average CV score: 0.67; average C score: 0.56; p=1).

There was no impact of access to the covered veranda on the feed (final mean feed consumption: CV treatment group: 3.05 kg/bird; C treatment group: 3.04 kg/bird; p=0.50) or water consumption (final mean water consumption: CV treatment group: 5.57 L/bird; C treatment group: 5.63 L/bird; p=0.88).

### Activity level and resting behavior

Only the location (inside CV rooms, inside C rooms, or covered verandas) was significant for running, walking, and lying behaviors (p<0.001). Broilers in the CV treatment group (mean of behaviors noted in the covered verandas and inside the CV rooms) expressed more walking and running behaviors than those in the C group (Table 5). There were more lying broilers counted in the C treatment group than in the CV group. However, there was no significant difference between the indoor CV rooms and C rooms. More walking and running behaviors were observed in the outdoor covered verandas than in the indoor CV rooms. There were more lying birds in the CV rooms than in the covered verandas.

**Table 5:** Mean (± standard deviation) of the standardised occurrence rates for walking and running behaviors and percentages of lying birds in covered verandas, inside the CV rooms, on average in the CV treatment group (average covered verandas and indoors) and in the C rooms.

	Covered verandas	CV rooms Inside	CV treatment (average covered verandas and inside)	C rooms	Significance of effects (p)		
					Indoors vs. outdoors (CV)	CV vs. C (indoors)	CV treatment vs. C
Walking standardised occurrence rate (per 100 broilers)	69.75 (27.55)	16.02 (12.32)	43.65 (15.84)	8.91 (5.74)	<0.001	0.55	<0.001
Running standardised occurrence rate (per 100 broilers)	88.66 (60.48)	0.87 (1.80)	44.88 (30.48)	0.64 (1.17)	<0.001	1	<0.001
Percentage of lying broilers	75.99 (17.25)	97.16 (3.42)	86.62 (9.34)	95.47 (4.73)	<0.001	0.84	0.001

### Use of covered verandas

**Fig. 2:**
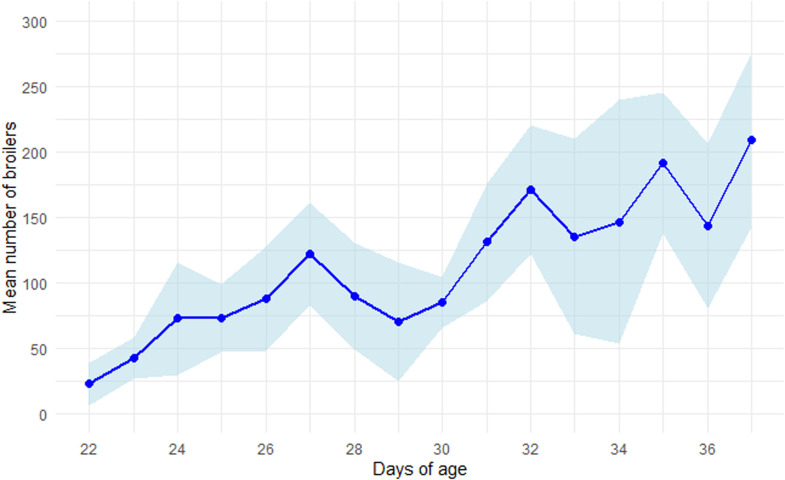
Mean number of broilers (± standard deviation, shown in light blue) counted per day of age in 36 m² of covered veranda. For each day, the values represent the mean of all hourly observations from 08:00 to 20:00, averaged across the three covered verandas.

**Fig. 3:**
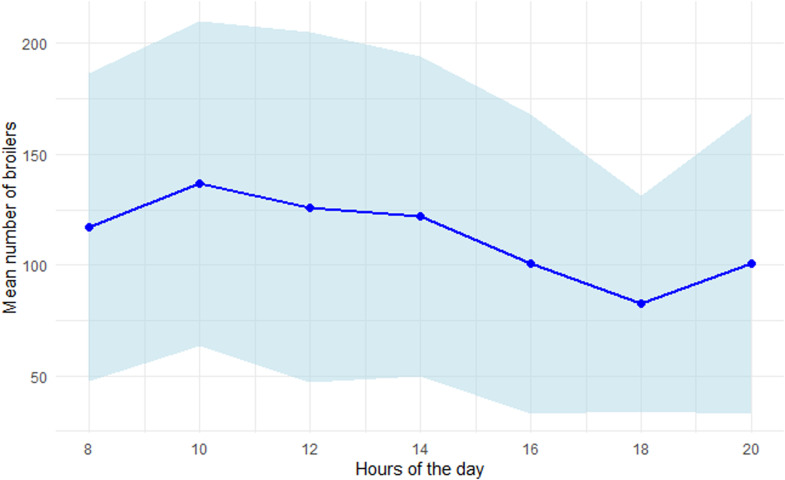
Mean number of broilers (associated standard deviation in light blue) counted in 36 m² of covered verandas by hour of the day, all days of age considered (mean of three covered verandas).

**Fig. A2:**
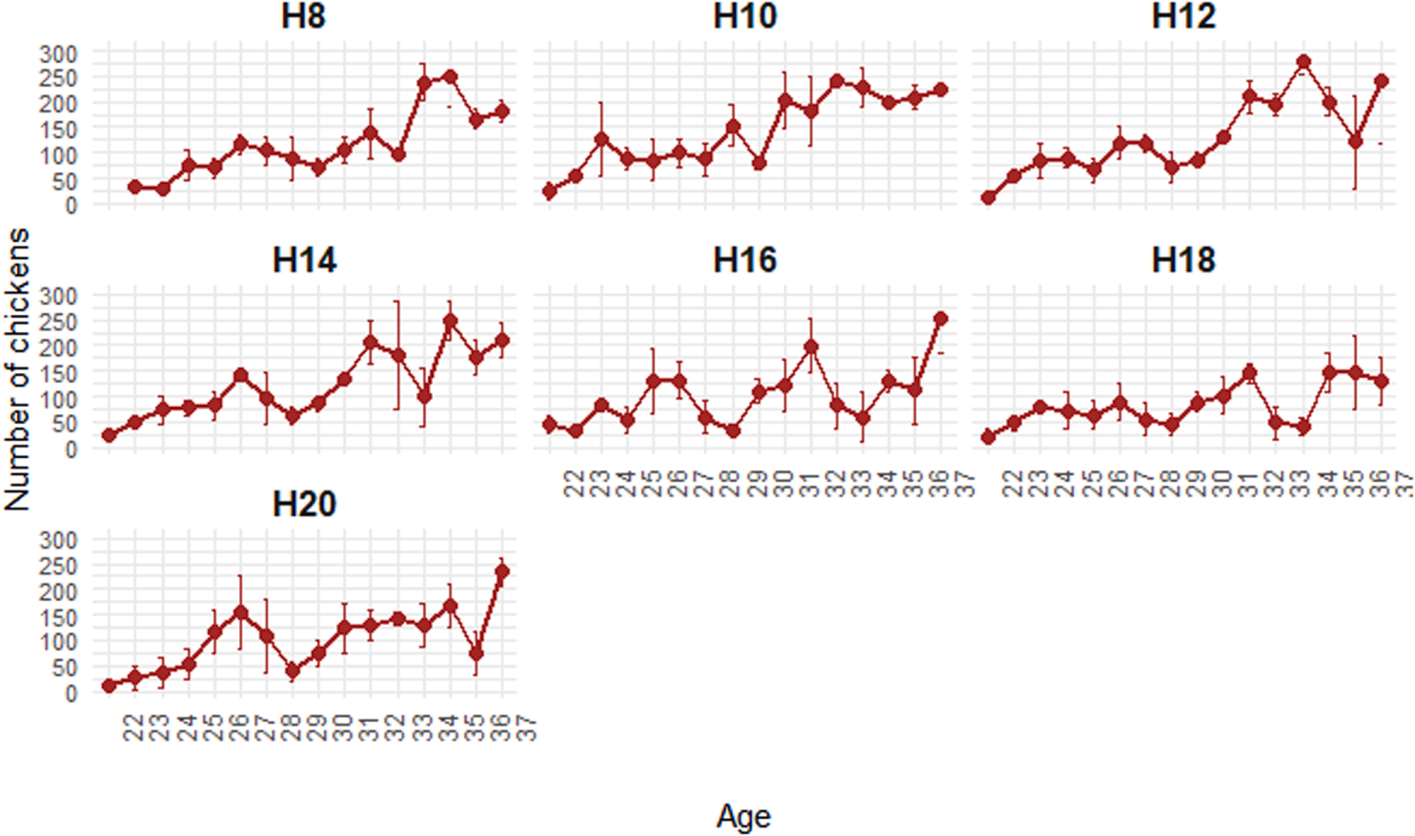
Mean number of broilers in 36 m² of covered verandas by age at each hour of the day from 08:00 to 20:00 (mean of three covered verandas and associated standard deviation).

**Fig. A3:**
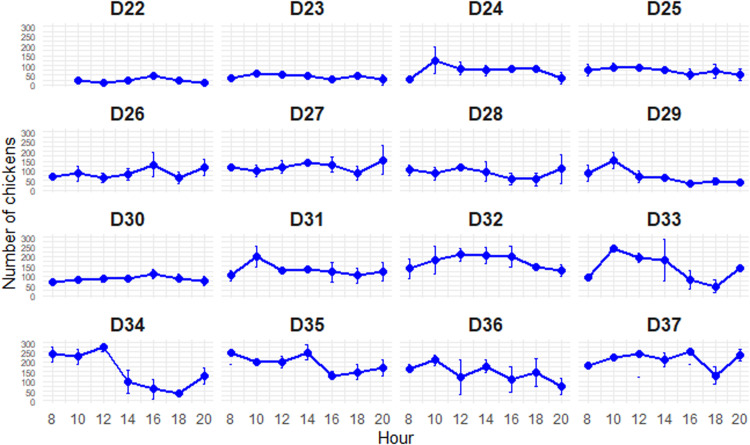
Mean number of broilers in 36 m² of covered verandas by hour of the day for each day of age from D22 to slaughter (mean of three covered verandas and associated standard deviation).

As the broilers aged, they were increasingly present in the covered verandas (regardless of the hour of day) (p<0.001) (Fig. 2, complementary data in Appendix Fig. A2). However, the broilers made significantly less use of the covered verandas as the day progressed, regardless of their age (p<0.001) (Fig. 3 and Appendix Fig. A3). The maximum number of broilers counted in the 36 m² covered veranda was reached around 10:00, before slowly decreasing until it reached the lowest number at 18:00. From 18:00 to 20:00, the number of broilers counted began to increase again.

## Discussion

The aim of this study was to evaluate the impact of covered verandas on the health and welfare of broilers and on zootechnical indicators. Access to the covered verandas did not impact either negatively or positively broiler weight or mortality during the rearing period. Access to the covered veranda was found to have no impact on feed and water consumption, even though covered verandas stimulated the birds’ activity in this area.

Broilers in our study were more active in the covered veranda than inside the rooms (exhibiting more walking and running behaviors). These findings are consistent with previous studies: increased activity was similarly observed in outdoor runs by Weeks et al. (1994) and Ruis et al. (2004), while Bergmann et al. (2017) reported comparable results in covered verandas. This suggests that access to an outdoor-like environment, whether fully open or roofed, can promote more active behaviors in broilers. Overall, broilers with access to a covered veranda (CV group) displayed more walking and running behaviors and were observed lying down less than those in the control group (C). These differences were mainly observed in the covered veranda, as no substantial behavioral differences were found between indoor rooms. Several hypotheses could be proposed to explain these results. Firstly, the birds observed in the covered verandas may represent only the most active and healthy individuals in the flock, with the fewest leg problems; they are therefore most prone to move further, leaving less active broilers indoors. However, no broilers in either group exhibited gait issues. One possible explanation is that the greater space available in the covered veranda allowed the broilers to move more freely, resulting in the more active behaviors observed, as a lack of sufficient individual space may lead to restricted movement (EFSA, 2023). In our study, based on the observed number of broilers counted on half the surface area of the covered verandas, we estimate a maximum stocking density of 13.2 kg/m² in the entire covered veranda at the end of the rearing period, while the remaining stocking density inside the CV rooms was 22 kg/m². Although these stocking densities could be considered low in comparison with commercial farms, the difference in stocking density between the covered veranda and the indoors may have helped stimulate birds' activity in the covered verandas.

Nonetheless, lower density in the covered veranda may not be the only possible contributor. Another explanation for the increased broiler activity in covered verandas could be natural light and its intensity. While both the covered verandas and indoor rooms (through several windows) provided natural light, the difference in activity levels between the two environments suggests that the intensity of natural light may play a more significant role than its mere presence, which is already known to stimulate bird activity (Bailie et al., 2013; de Jong and Gunnink, 2019). Studies on artificial light intensity have shown that dimmer light is associated with inactive behaviors (e.g. resting), while brighter light encourages more active behaviors, such as preening, foraging, or litter-directed active behaviors (Davis et al., 1999; Alvino et al., 2009; Deep et al., 2010; van der Eijk et al., 2025). This supports the idea that natural light intensity, like artificial light, might play a key role in influencing broiler behavior. However, in these studies, locomotor behaviors were not, or hardly, impacted by artificial light intensity. Nevertheless, studies on environmental enrichments and natural light have shown an increased activity, including locomotor behaviors, when natural light was added to enriched rearing conditions (Bailie et al., 2013; de Jong and Gunnink, 2019). If we consider the covered verandas as an enriched environment offering various sensory stimulations — such as fresh air, variations in odour and temperature, or direct sunlight — our results may align with those of Bailie et al. (2013) and de Jong and Gunnink (2019). This is particularly notable given that the indoor rearing environment was already enriched with elevated platforms, alfalfa bales and natural light.

Thus, the higher activity level of broilers in the covered veranda than indoors may be explained by the lower stocking density, brighter natural light, and/or other outdoor sensory stimulations. Lastly, studies in other species, such as great apes (Kurtycz et al., 2014), have shown more positive affective states when given the choice between indoor and outdoor environments than when no choice was given. And Ross (2006) demonstrated that providing access to a private space (off-exhibit holding space in a zoo) improved welfare in polar bears, even when it was rarely used, suggesting that the provision of choice was more impactful than the use of this private space itself. While these studies focused on very different species, the principle of offering choice may, to some extent, be extrapolated to poultry and to the benefits of access to a covered veranda. However, the present study lacks the data needed to reach a conclusion on this hypothesis (i.e. that choice may have a positive impact on affective states and welfare). Further research is needed to assess the affective states of broilers and other species given the option to move between indoor and outdoor environments, as there is a gap of knowledge on this topic.

The broilers in our study increasingly used the covered verandas as they got older, as already shown in Bergmann’s study (2017). Bergmann et al. hypothesised that cautiousness (avoiding predators) played a role during the first days of access to the covered veranda, which was later replaced by possible habituation. Although opposite results (i.e. older birds used the covered veranda less) could have been anticipated and attributed to a general decrease in activity with age in broilers, our study confirms Bergmann’s findings with a different genetic strain. Weather conditions could also have influenced use by broilers of the covered verandas in our study. Although we collected general meteorological data from a local weather station (outdoor temperatures ranging from 1.8°C to 18.6°C), we did not record the temperature and humidity inside the covered verandas, which prevented direct analysis of its effect. The local weather station data provided an indication of the general weather conditions during our observations; however, they are not fully suitable for further analysis because the conditions recorded were likely to be too different from those actually experienced by the broilers. Previous studies (e.g., Bergmann et al., 2017) have shown that higher outdoor temperatures tend to increase broiler use of outdoor areas. Therefore, while we cannot evaluate this factor precisely in our study, it is likely that weather conditions contributed to variability in veranda use. Future research should focus on the effects of season, temperature, and humidity on both the use of covered verandas by the birds and their associated impacts.

In the present study, the number of birds in the covered verandas was recorded throughout the day, at two-hour intervals from 08:00 to 20:00. According to our statistical model, more broilers were observed in the covered verandas in the early hours of the day than later on, regardless of their age. However, this result is not clearly visible in Fig. 3, likely due to the considerable standard deviations caused by averaging the mean number of broilers across days of age, from 22 to 37 days. Nevertheless, the daily variation in the number of broilers in the covered veranda may be associated with their rhythm of activity throughout the day. Broilers exhibit a biphasic activity pattern, with peaks in the morning and evening, which develops in young chicks and persists into adulthood (Bessei et al., 2023). This could explain the higher number of broilers in the covered verandas in the morning, where they were more active than when in the rooms. However, the expected evening peak in activity may not have been captured in our observations if it occurred after 20:00, beyond the recording schedule and close to sunset (at this period of the year, sunset occurred between 20:45 and 21:10). Occupation of the covered verandas could also be related to the sunshine: as the sun rises in the east and the covered verandas face south-east, there was more sunshine earlier in the day (at this period, sunrise occurred between 07:10 and 06:35) than at the end.

Broilers with access to covered verandas had a lower prevalence of hock burns compared to those in the control group, while no broilers in either group showed signs of footpad dermatitis or gait issues. Both hock burns and footpad dermatitis are multifactorial conditions but are primarily associated with poor litter quality (EFSA, 2023). In this study, the litter quality indoor was good (mean score<1) and did not differ between the treatments, ruling out litter condition as the cause of the observed hock burns. Likewise, although not noted, the litter quality in the covered veranda was good (personal observation). According to Haslam et al. (2006), as the plantar surface of the foot is in contact with the litter both when the bird is active and resting, footpad dermatitis is more closely linked to litter quality than are hock burns. Conversely, hock burns are more indicative of prolonged lying behavior, which reflects inactivity. Thus, increased broiler activity reduces the risk of hock burns by minimising contact between the hocks and the litter (de Jong, et al., 2016). This aligns with our findings, as broilers in the C treatment group were more frequently observed in a lying position than those with access to covered verandas, and had a higher prevalence of hock burns.

Stocking density may also influence foot and hock lesions (Knierim, 2013; Mocz et al., 2022), but this effect occurs indirectly, mainly through factors such as degradation of the rearing environment — particularly litter quality (Dawkins et al., 2004; EFSA, 2023) — and restricted movement, which increases the time spent on suboptimal litter. In this study, stocking densities were relatively low. For the CV treatment group, the final stocking density, considering both the indoor area and the covered veranda, was approximately 18.6 kg/m². For the C treatment group, it reached 26 kg/m² at the end of the rearing period. Neither of the densities appeared to affect litter quality. The increased activity observed in broilers with access to covered verandas appears to be the main contributing factor to the reduction in hock burns observed in the CV treatment group.

## Conclusion

Giving access to a covered veranda brought welfare benefits such as better hock condition and more active behaviors. Nonetheless, no zootechnical indicators (body weight, feed and water consumption, mortality, litter quality) were impacted. While the construction of a covered veranda represents a substantial investment for farmers, this study suggests that covered veranda access may bring significant welfare improvements for the birds. However, it is important to note that the observed improvements in welfare should be considered within the context of the medium-growing broiler strain used in this study and the relatively early slaughter age, as well as the controlled experimental rearing conditions. This topic deserves further research to better understand how access to a covered veranda impacts broiler welfare.

## Disclosures

The authors declare that they have no known competing financial interests or personal relationships that could have appeared to influence the work reported in this paper.

## Declaration of competing interest

The authors declare that they have no known competing financial interests or personal relationships that could have appeared to influence the work reported in this paper.
